# Simulated Identification of Silent COVID-19 Infections Among Children and Estimated Future Infection Rates With Vaccination

**DOI:** 10.1001/jamanetworkopen.2021.7097

**Published:** 2021-04-23

**Authors:** Seyed M. Moghadas, Meagan C. Fitzpatrick, Affan Shoukat, Kevin Zhang, Alison P. Galvani

**Affiliations:** 1 Agent-Based Modelling Laboratory, York University, Toronto, Ontario, Canada; 2 Center for Vaccine Development and Global Health, University of Maryland School of Medicine, Baltimore; 3 Center for Infectious Disease Modeling and Analysis, Yale School of Public Health, New Haven, Connecticut; 4 Faculty of Medicine, University of Toronto, Toronto, Ontario, Canada

## Abstract

**Importance:**

A significant proportion of COVID-19 transmission occurs silently during the presymptomatic and asymptomatic stages of infection. Children, although important drivers of silent transmission, are not included in the current COVID-19 vaccination campaigns.

**Objective:**

To estimate the benefits of identifying silent infections among children as a proxy for their vaccination.

**Design, Setting, and Participants:**

This study used an age-structured disease transmission model, parameterized with census data and estimates from published literature, to simulate the estimated synergistic effect of interventions in reducing attack rates during the course of 1 year among a synthetic population representative of the US demographic composition. The population included 6 age groups of 0 to 4, 5 to 10, 11 to 18, 19 to 49, 50 to 64, and 65 years or older based on US census data. Data were analyzed from December 12, 2020, to February 26, 2021.

**Exposures:**

In addition to the isolation of symptomatic cases within 24 hours of symptom onset, vaccination of adults was implemented to reach a 40% to 60% coverage during 1 year with an efficacy of 95% against symptomatic and severe COVID-19.

**Main Outcomes and Measures:**

The combinations of proportion and speed for detecting silent infections among children that would suppress future attack rates to less than 5%.

**Results:**

In the base-case scenarios with an effective reproduction number *R*_e_ = 1.2, a targeted approach that identifies 11% of silent infections among children within 2 days and 14% within 3 days after infection would bring attack rates to less than 5% with 40% vaccination coverage of adults. If silent infections among children remained undetected, achieving the same attack rates would require an unrealistically high vaccination coverage (≥81%) of this age group, in addition to 40% vaccination coverage of adults. The estimated effect of identifying silent infections was robust in sensitivity analyses with respect to vaccine efficacy against infection and reduced susceptibility of children to infection.

**Conclusions and Relevance:**

In this simulation modeling study of a synthetic US population, in the absence of vaccine availability for children, a targeted approach to rapidly identify silent COVID-19 infections in this age group was estimated to significantly mitigate disease burden. These findings suggest that without measures to interrupt transmission chains from silent infections, vaccination of adults is unlikely to contain the outbreaks in the near term.

## Key Points

**Question:**

Is a targeted strategy for identification of silent COVID-19 infections among children in the absence of their vaccination associated with reduced infection rates in the general population?

**Findings:**

In this simulation modeling study, identifying 10% to 20% of silent infections among children within 3 days after infection would bring attack rates below 5% if only adults were vaccinated. If silent infections among children remained undetected, achieving the same attack rate would require an unrealistically high vaccination coverage (≥81%) of this age group, in addition to vaccination of adults.

**Meaning:**

These findings suggest that rapid identification of silent infections among children may achieve comparable effects as would their vaccination.

## Teaser

This simulation modeling study estimates the benefits of identifying silent COVID-19 infections among children as a proxy for their vaccination.

## Introduction

The ongoing COVID-19 pandemic has caused significant global morbidity and mortality.^1^ Public health interventions, including social distancing, testing, contact tracing, and isolation of cases, have substantially reduced the spread of SARS-CoV-2.^2,3,4^ However, enhanced viral transmissibility due to the emergence of novel variants^5,6,7,8^ and the erosion of support for prolonged mitigation measures have raised concerns about perpetual waves of COVID-19 outbreaks.

Global efforts to ameliorate the impact of this deadly disease have galvanized the development of a number of vaccines that have received emergency use authorization from regulatory bodies in several countries,^9^ including the Pfizer-BioNTech and Moderna vaccines in the US.^10,11^ Most clinical trials have followed US Food and Drug Administration guidelines,^12^ prioritizing the evaluation of vaccine safety and efficacy in adults, because this population group has borne most of the reported infections, severe illnesses, and deaths.^13,14,15^ Given the lack of vaccine safety and efficacy data for children,^16^ vaccination campaigns have been targeted toward adults (aged ≥18 years) and those at high risk of infection and severe outcomes. Thus, nonpharmaceutical interventions will still be required for mitigating disease transmission among children.

Given that children are more likely to develop asymptomatic infection compared with other age groups,^17,18,19,20^ they can be important drivers of silent transmission.^21^ We developed an age-stratified SARS-CoV-2 transmission model to estimate the impact of a targeted strategy for identifying silent infections among this age group when only adults are vaccinated (eMethods and eFigure 1 in the Supplement). We then calculated the proportion and the speed of identification required to suppress future attack rates to less than 5% and, alternatively, the vaccination coverage among children that could achieve the same goal.

## Methods

### Model Structure

This simulation modeling study used publicly available data and parameter estimates from previously published studies and did not require ethics review or approval. We modeled the transmission of SARS-CoV-2 by developing an age-structured compartmental model that accounted for the natural history of disease as well as self-isolation and vaccination dynamics (eTable 1 in the Supplement). The population was stratified into 6 age groups: 0 to 4, 5 to 10, 11 to 18, 19 to 49, 50 to 64, and 65 years or older, parametrized from US census data.^22^ Model parameterization was based on age-specific data regarding asymptomatic rates of infection and relative transmissibilities during different stages of infection.^23,24^ Contact rates between and within age groups were heterogeneous and derived from empirical studies of social mixing.^25,26^ Newly infected individuals moved from the susceptible stage to the latent stage and proceeded to a communicable silent infection stage (ie, either asymptomatic or presymptomatic). A proportion of infected individuals remain asymptomatic until recovery,^17,18,19,20^ whereas others develop symptoms after the presymptomatic stage. The mean duration of these epidemiological stages and other age-specific relevant parameters are derived from publicly available sources and published estimates (eTable 2 in the Supplement). For the base case, susceptibility to infection was constant across ages, but as a sensitivity analysis, we reduced susceptibility by half for children younger than 10 years.^27,28,29^

In our model, all symptomatic cases were identified and isolated within 24 hours after symptom onset. For isolation of silent infections, we varied the proportion identified and the time from infection to identification in the range 2 to 5 days, reflecting observed delays in testing and contact tracing. Isolated individuals limited their daily contacts to the age-specific rates reported during COVID-19 lockdown^25,26^ until the end of their infectious period.

In vaccination scenarios, we distributed vaccines over time among individuals older than 18 years from the onset of simulations. Given vaccine prioritization of high-risk groups, we assumed that 80% of individuals 50 years and older and 22% of adults aged 18 to 49 years would be vaccinated, resulting in an overall vaccine coverage of 40% among adults within 1 year.^30^ We then extended our analysis for vaccination coverages of adults to 60%. The vaccine efficacy against developing symptomatic or severe disease after vaccination was 95%, based on the results of phase 3 clinical trials.^31,32^ We also assumed that vaccine efficacy against infection was 50% lower than the efficacy against disease, but also considered a scenario with the same efficacy of 95% as a sensitivity analysis (eResults 2 and eFigure 4 in the Supplement).

We calibrated the transmission rate to an effective reproduction number *R*_e_ = 1.2, accounting for the effect of current nonpharmaceutical interventions and 10% preexisting immunity in the population.^33^ To capture the age distribution of preexisting population immunity, the outbreak was simulated to the time before vaccination. The age-specific infection rates were then derived when the overall attack rate reached 10%,^34,35,36^ corresponding distributions of which were used as the starting population for the vaccination model. We assumed that the transmission rate was identical for presymptomatic and symptomatic cases but reduced by a mean of 74% for asymptomatic cases based on recent estimates of asymptomatic COVID-19 infectivity,^24^ and that recovered individuals were not susceptible to reinfection. We then conducted model simulations independently for each intervention scenario and calculated the attack rate as the proportion of the population infected within 1 year. For the scenario without vaccination, we considered identification of silent infections among all age groups. When vaccination of adults was implemented, identification of silent infections was targeted toward only children with delays of 2 to 5 days after infection. In this targeted approach, the proportion and the speed of identification required to suppress future attack rates to 5% were determined. In the absence of preexisting immunity and vaccination, most populations experienced an attack rate in the range of 1% to 5% during the first wave of the COVID-19 pandemic. Therefore, we assumed that an attack rate of less than 5% would be a reasonable threshold to consider for our analysis in the presence of preexisting immunity and vaccination, whereas other nonpharmaceutical interventions are accounted for by the effective reproduction number. For each scenario of a time delay to identification, we calculated the vaccine coverage of children that would be required in addition to vaccination of adults to achieve a similar attack rate if efforts to identify silent infections were completely halted.

### Statistical Analysis

Simulations were conducted from December 12, 2020, to February 26, 2021. Simulations were seeded with an initial case in each age group in the latent stage in a population of 10 000 individuals for a time horizon of 1 year. In each scenario, mean cumulative infections were calculated for 1000 independent replications with disease-specific parameters sampled from their respective distributions (eTable 2 in the Supplement). Credible intervals (CrIs) at the 5% significance level were generated using the bias-corrected and accelerated bootstrap method (with 500 replications).

## Results

### Identification of Silent Infections in the Population

**Figure 1.**
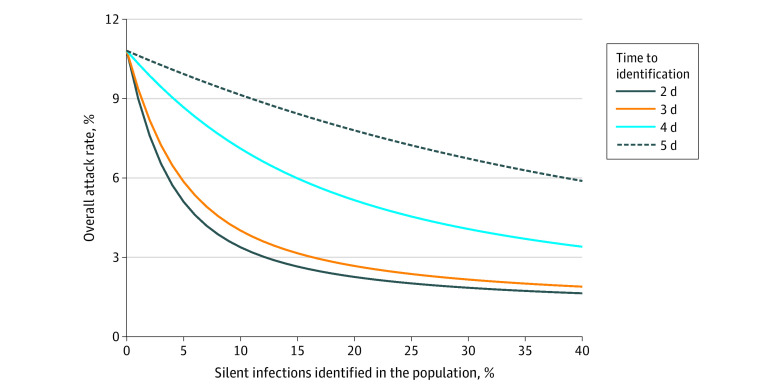
Estimated Mean Attack Rates Without Vaccination and With Identification of Silent Infections in the Population Color curves represent attack rates when different proportions of silent infections are identified in the population, corresponding to different time delays after infection.

In the absence of vaccination and with *R*_e_ = 1.2, an overall attack rate of 10.8% (95% CrI, 10.5%-11.2%) would be expected when no silent infections in the population are detected (Figure 1). If silent infections are identified within 2 or 3 days after infection, a rapid decline in the attack rate can be achieved with isolation of a relatively small (≤15%) proportion of silent infections, with diminishing returns as identification rates rise to greater than 20% (Figure 1). However, with a further delay in identification, a significantly larger proportion of silent infections needs to be detected to have a similar impact in reducing the attack rate. For instance, with 10% of silent infections identified in the population and isolated within 2 days of infection, the attack rate can be reduced to 3.4% (95% CrI, 3.2%-3.5%). To achieve the same mean attack rate with a delay of 3 days, a detection rate of 13% for silent infections would be required; a delay of 4 days, a detection rate of 42%; and a delay of 5 days, a detection rate of 98%.

### Targeted Identification of Silent Infections Among Children

**Figure 2.**
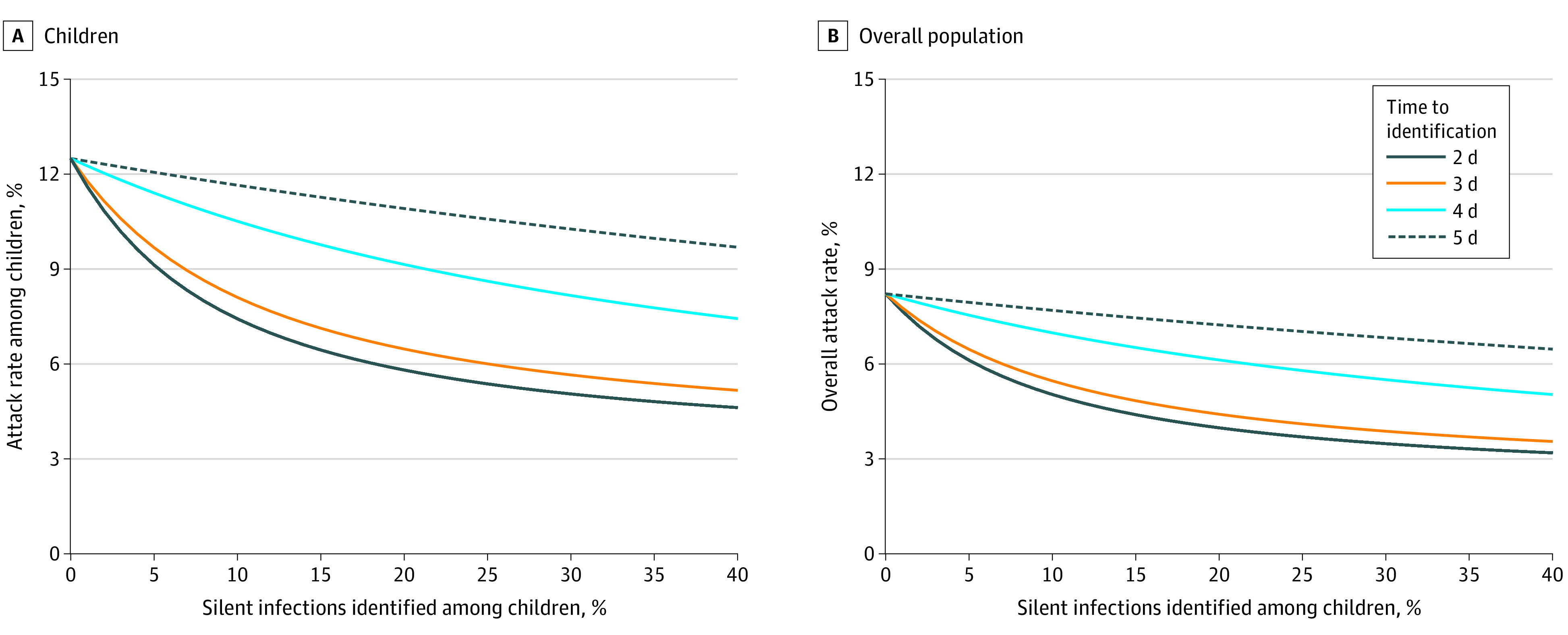
Estimated Mean Attack Rates With Vaccination of Adults and Identification of Silent Infections Among Children A, Attack rates among children younger than 18 years. B, Attack rates among the entire population. Color curves represent attack rates when different proportions of silent infections are identified in children, corresponding to different time delays after infection. Vaccination coverage of adults reached 40% within 1 year.

With vaccines distributed to only adults, estimated attack rates would be reduced to 12.5% (95% CrI, 11.9%-13.2%) among children and 8.2% (95% CrI, 7.8%-8.9%) among the overall population without identification of silent infections (Figure 2). We simulated the effect of a targeted strategy for identification of silent infections only among children on reducing attack rates. Attack rates declined rapidly with increasing identification of silent infections within 2 or 3 days after infection (Figure 2). For example, identification of at least 11% within 2 days and 14% within 3 days would suppress the overall attack rate to less than 5% (Figure 2B). With a delay of 4 days, an identification rate of 41% (a 3.7-fold increase) compared with a 2-day delay is needed to bring attack rates to less than 5%. With a delay of 5 days, an identification rate of 97% (a 6.9-fold increase compared with a 3-day delay in identification) is needed to bring attack rates to less than 5%. If silent infections among children remained undetected, an unrealistically high vaccination coverage (≥81%) of this age group, in addition to 40% vaccination coverage of adults, must be achieved within 1 year to suppress attack rates to less than 5%. These results suggest that, even when vaccines become available for children, rapid identification of their silent infections is still essential to mitigate disease burden in the population.

**Figure 3.**
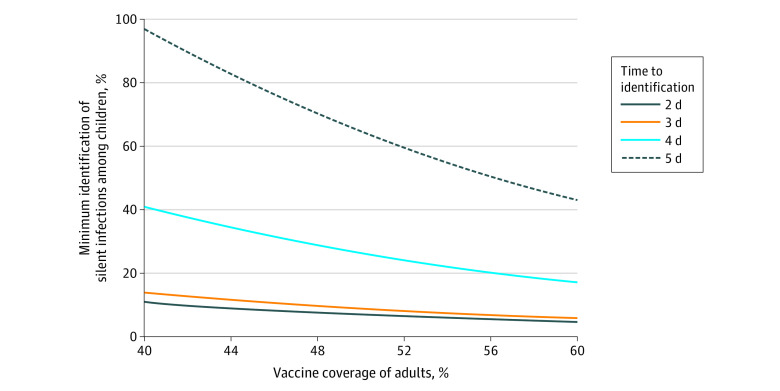
Required Identification of Silent Infections Among Children to Reduce Attack Rates to Less Than 5% With Vaccination of Adults Color curves represent the minimum identification level of silent infections among children required to suppress the overall attack rate to less than 5%, with different vaccination coverage of adults and time delays for identification after infection.

We further simulated the model to determine the estimated effect of vaccine coverage on the minimum level of silent infections required to be identified among children to suppress the overall attack rate in the population to less than 5%. We found that when vaccination coverage of adults is expanded from 40% to 60%, the minimum identification levels dropped from 11% with a delay of 2 days to 5% and from 14% with a delay of 3 days to 6% (Figure 3). When delays increased to 4 and 5 days, the minimum identification levels were 17% and 43%, respectively, for a 60% vaccine coverage of adults during a 1-year time-horizon, both of which were higher than those required for delays of 2 and 3 days with 40% coverage of adults.

### Sensitivity Analyses

We evaluated whether reduced susceptibility to infection among children or higher vaccine efficacy against infection would affect the results. If susceptibility among children younger than 10 years was reduced by half, then less contact tracing was necessary to control COVID-19 with vaccination of adults (eResults 1 and 2 and eFigures 1 and 2 in the Supplement). For instance, 5% identification of silent infections within 2 days after infection, 6% within 3 days, 19% within 4 days, or 47% within 5 days would suppress the overall attack rate to less than 5% (eFigure 2 in the Supplement), or alternatively, vaccination coverage among children would need to reach 73% within 1 year. We observed qualitatively similar results when vaccine protection against infection was the same as efficacy against disease (eResults 2 in the Supplement). In addition, we conducted sensitivity analyses for a higher reproduction number of *R*_e_ = 1.5 and for a lower reproduction number of *R*_e_ = 0.9 to account for other factors, such as seasonal effects that may influence disease transmissibility (eResults 3 and 4 and eFigures 5-7 in the Supplement). The results indicate that the identification of silent infections has a greater estimated effect on reducing attack rates as the reproduction number increases.

## Discussion

A substantial proportion of COVID-19 cases are attributed to silent transmission from individuals in the presymptomatic and asymptomatic stages of infection.^37,38,39,40^ Children are particularly likely to have mild or asymptomatic infections,^18,41^ increasing the likelihood that they will serve as unidentified links between more severe cases. Although vaccines against COVID-19 now have emergency use authorization, these products have not yet been tested in children, and it will be several months before children are widely vaccinated. In the absence of their vaccination, augmenting symptom-based screening with identification of silent infections is essential to control outbreaks.^23,42^ Our results suggest that the proportion of silent infections being identified among children is secondary to the speed of identification. For example, when *R*_e_ = 1.2, if the time from infection to identification was reduced from 4 to 2 days after infection without reduction of susceptibility for children younger than 10 years, the same overall attack rate of 5% could be achieved with identifying more than a 3.7-fold (from 41% to 11%) lower proportion of silent infections. Accelerating identification from 5 to 3 days corresponds to an estimated 6.9-fold (from 97% to 14%) reduction in the proportion for detection of silent infections among children required to suppress the overall attack rate. Therefore, enhancing the capacity for rapid tracing of contacts of symptomatic individuals is critical to mitigating disease transmission.

The resurgence of COVID-19 cases before initiating vaccination in December 2020 overwhelmed the health care system in many jurisdictions, hampering the ability of public health to conduct effective contact tracing.^43,44,45^ Vaccination can alleviate the burden of COVID-19 outbreaks and may allow for resource reallocation toward targeted contact tracing in settings where unvaccinated individuals congregate, such as schools and day-care facilities. In a scenario in which vaccines are only available for adults (with *R*_e_ = 1.2), our results show that if only 1 in 10 infected children were identified within 2 days after infection or 1 in 7 within 3 days after infection (eg, by contact tracing and routine testing), the overall attack rate could be reduced to less than 5%. With recent advances in noninvasive testing modalities, such as saliva tests,^46^ routine testing in settings such as schools could feasibly achieve this identification target.

### Limitations

Our results should be interpreted within the context of model limitations. First, we did not explicitly include the effects of nonpharmaceutical interventions, but instead calibrated the model to current estimates of the effective reproduction number that implicitly accounts for these effects.^33^ The relaxation of such measures would increase the need for vigilant contact tracing among unvaccinated populations. Given COVID-19 awareness and public health recommendations, we assumed that all individuals with symptomatic cases self-isolate within 24 hours of symptom onset. Despite this high rate of rapid self-isolation, our sensitivity analyses confirm that rapid contact tracing will still be an important dimension of control even if child susceptibility is half that of adults. With vaccination of adults, we evaluated the impact of identifying silent infections only among children. However, our results should not be interpreted as excluding adults for identification of silent infections. Our focus on targeting children is largely motivated by current deliberations regarding the reopening of schools and the potential for ensuing elevated spread of COVID-19 through asymptomatic infections in this population. Simultaneously expanding identification of silent infections among young adults currently not prioritized for vaccination would contribute to earlier control of outbreaks.

For the estimated effect of vaccination, we parameterized the model with results of phase 3 clinical trials for vaccine efficacy.^31,32^ Given the uncertainty around distribution capacity and uptake of vaccines, we simulated the model with a vaccination rate to achieve 40% to 60% vaccine coverage of adults within 1 year. If vaccines are distributed more rapidly or with higher uptake, it is possible that the rapid rise of population-level immunity could reduce the need for a targeted strategy to identify silent infections in children. However, given the current limitations in initial vaccine supplies and challenges with cold-chain distribution of messenger RNA vaccines,^47,48^ it is unlikely that vaccination will remove the need for nonpharmaceutical interventions in the near term.

## Conclusions

In this simulation modeling study of COVID-19 transmission dynamics, identification of silent infections among children was shown to be an important strategy as vaccination campaigns continue to immunize adults. We found that early interruption of transmission chains is critical to outbreak control. Contact tracing at the time of symptom onset or testing, as opposed to at the time of testing results, could have an important impact on suppressing onward disease transmission by asymptomatic or presymptomatic infections, especially in the context of delays in turnaround time for COVID-19 test results.

## Notes

Supplement. **eMethods.** The Model **eFigure 1.** Schematic Model Diagram for Disease Transmission Dynamics **eTable 1.** Description of the Model State Variables **eTable 2.** Description of the Model Parameters and Their Associated Values **eResults 1.** *R*_e_ = 1.2 and Reduced Susceptibility of Children **eFigure 2.** Estimated Mean Attack Rate Achieved With Different Rates of Silent Infections (ie, Asymptomatic and Presymptomatic) Identified and Isolated Among Children, When Only Adults Were Vaccinated **eFigure 3.** Minimum Identification Level of Silent Infections Among Children (y-axis) Required to Bring the Overall Attack Rate in the Population Below 5% as a Function of Vaccine Coverage of Adults With Different Delays in Identification Post Infection **eResults 2.** *R*_e_ = 1.2 and 95% Vaccine Efficacy Against Infection **eFigure 4.** Estimated Mean Attack Rate Achieved With Different Rates of Silent Infections (ie, Asymptomatic and Presymptomatic) Identified and Isolated Among Children, When Only Adults Were Vaccinated **eResults 3.** *R*_e_ = 1.5 **eFigure 5.** Estimated Mean Attack Rate in the Population Achieved With Different Rates of Silent Infections (ie, Asymptomatic and Presymptomatic) Identified and Isolated in the Population Without Vaccination **eFigure 6.** Estimated Mean Attack Rate Achieved With Different Rates of Silent Infections (ie, Asymptomatic and Presymptomatic) Identified and Isolated Among Children, When Only Adults Were Vaccinated **eFigure 7.** Estimated Mean Attack Rate Achieved With Different Rates of Silent Infections (ie, Asymptomatic and Presymptomatic) Identified and Isolated Among Children, When Only Adults Were Vaccinated **eResults 4.** Reduced Reproduction Number: *R*_e_ = 0.9 **eReferences.**
